# Expectations of the timing and intensity of a stimulus propagate to the auditory periphery through the medial olivocochlear reflex

**DOI:** 10.1093/cercor/bhac002

**Published:** 2022-01-30

**Authors:** Sho Otsuka, Seiji Nakagawa, Shigeto Furukawa

**Affiliations:** Center for Frontier Medical Engineering, Chiba University, Chiba, Japan; NTT Communication Science Laboratoires, NTT Corporation, Kanagawa, Japan; Center for Frontier Medical Engineering, Chiba University, Chiba, Japan; NTT Communication Science Laboratoires, NTT Corporation, Kanagawa, Japan

**Keywords:** auditory periphery, efferent system, temporal expectation, top-down control

## Abstract

Expectations concerning the timing of a stimulus enhance attention at the time at which the event occurs, which confers significant sensory and behavioral benefits. Herein, we show that temporal expectations modulate even the sensory transduction in the auditory periphery via the descending pathway. We measured the medial olivocochlear reflex (MOCR), a sound-activated efferent feedback that controls outer hair cell motility and optimizes the dynamic range of the sensory system. MOCR was noninvasively assessed using otoacoustic emissions. We found that the MOCR was enhanced by a visual cue presented at a fixed interval before a sound but was unaffected if the interval was changing between trials. The MOCR was also observed to be stronger when the learned timing expectation matched with the timing of the sound but remained unvaried when these two factors did not match. This implies that the MOCR can be voluntarily controlled in a stimulus- and goal-directed manner. Moreover, we found that the MOCR was enhanced by the expectation of a strong but not a weak, sound intensity. This asymmetrical enhancement could facilitate antimasking and noise protective effects without disrupting the detection of faint signals. Therefore, the descending pathway conveys temporal and intensity expectations to modulate auditory processing.

## Introduction

Humans constantly generate predictions about future events and adaptively optimize neural processing in order to cope with a large amount of information with limited neural resources (Rao and Ballard 1999; Friston 2005; Palmer et al. 2015; Singer et al. 2018). Such predictions not only pertain to what will happen in the sensory environment but also concern the time at which the upcoming event will occur (reviewed in Arnal and Giraud 2012; Nobre and van Ede 2018; Rimmele et al. 2018). Temporal expectations guide the dynamical peak of attention to a particular time point and allow for the allocation of processing resources solely to relevant sensory events, thus enabling faster responses and improved behavioral performance (Niemi and Lehtonen 1982; Barnes and Jones 2000; Correa et al. 2005; Rohenkohl et al. 2012; Cravo et al. 2013).

Recent human studies have started to uncover the brain regions underlying temporal expectation. Functional brain imaging has pointed toward the involvement of multiple neocortical regions responsible for cognitive processing, namely the prefrontal cortex (Coull et al. 2000; Vallesi et al. 2009), left-lateralized parietal cortex (Coull and Nobre 1998; Coull et al. 2008), and ventral premotor cortex (Schubotz 2007; Coull et al. 2008). In addition, the integration of supplementary motor areas and the superior temporal gyrus has been suggested (Cui et al. 2009). Consistently, the increased response to stimuli occurring at expected time points is observed in the P300 wave and the lateralized readiness potential, both of which are linked to response preparation and execution after the appearance of target events (Miniussi et al. 1999; Griffin et al. 2002; Los and Heslenfeld 2005; Correa et al. 2006; Hackley et al. 2007). Sensory processing was also reported to be modulated by temporal expectation in perceptual potentials originating in the auditory and visual cortex (Ghose and Maunsell 2002; Jaramillo and Zador 2011), as well as in auditory subcortical areas (Gorina-Careta et al. 2016). With respect to resource allocation, it is reasonable that such expectation-based optimization begins at early processing stages. In visual pathways, for example, even oculomotor behavior, that is, saccades, ocular drift, and blinks, is reported to be modulated by temporal expectation (Betta and Turatto 2006; Dankner et al. 2017; Amit et al. 2019). Furthermore, the pupil light reflex (PLR), which changes the pupil size and adjusts the amount of light entering the eye to balance sensitivity and visual acuity (Binda and Gamlin 2017), is modulated by the expectation of luminance coming from the point in space toward which gaze is moving (Mathôt et al. 2015). This would allow the periphery to prepare for intense luminance and achieve an optimal sensitivity for upcoming stimuli under a limited dynamic range.

As for the auditory pathway, however, the location where the modulation according to temporal expectations first occurs, as well as its role in such modulatory process, is unknown. As in the visual system, the information concerning temporal expectations may modulate the auditory periphery through the top-down control of peripheral receptor activity via the descending pathway. This could be accomplished through two major efferent feedback pathways (Liberman and Guinan 1998), the middle ear muscle reflex (MEMR) and the medial olivocochlear reflex (MOCR), both of which can decrease responses at the auditory periphery. The MEMR acts by stiffening the ossicle chains (Mukerji et al. 2010), whereas the MOCR induces an inhibitory effect on the motility of outer hair cells (OHCs; Guinan 2006). The MOCR and MEMR can be considered the auditory counterparts of the PLR, as many similarities exist between these reflexes, including their brainstem origination aimed at optimizing sensory dynamic range and their slow response speed (operating over hundreds of milliseconds) (Liberman and Guinan 1998; Backus and Guinan 2006; Guinan 2006; Binda and Gamlin 2017). Similar to the PLR, which receives descending projections from the cortical area (Ebitz and Moore 2017), there is some evidence of corticofugal projections to MOC neurons via subcortical nuclei (Terreros and Delano 2015) and top-down controls of the MOCR (Dragicevic et al. 2015). Specifically, modulation of the MOCR is achieved by orienting attention to a specific laterality (Froehlich et al. 1993), auditory target (Smith et al. 2012), frequency (Maison et al. 2001), working memory task (Marcenaro et al. 2021), or visual task (Delano et al. 2007; Wittekindt et al. 2014). Dragicevic et al. (2019) further provided evidence of the interaction between otoacoustic emissions (OAEs) and low-frequency cortical oscillations during selective attention, which supports the possibility that cognitive processing at cortical levels can modulate the MOCR via the corticofugal pathways. In addition, the stapedius muscle can be activated even without acoustic stimulation during (and in anticipation of) vocalization to reduce self-stimulation (Borg and Zakrisson 1975), and some individuals can voluntarily engage the MEMR. Although this voluntary control of the MEMR is expected to be attributed to the descending projections from the cerebral cortex to stapedius motoneurons, no direct evidence of this has been provided (Mukerji et al. 2010). Given the abundant evidence for corticofugal projections to MOC neurons, we hypothesized that anticipatory top-down MOCR control is plausible.

Furthermore, analogous to the PLR-mediated changes in eye movements as a result of changes in luminance, expectations about the intensity of upcoming sounds are also be important for the MOCR and are combined with temporal expectation for optimal responses. The MOCR-induced inhibition of the OHC amplification improves signal detection in noise by preventing auditory nerve adaptation to the noise (Kawase and Liberman 1993; Kumar and Vanaja 2004; Otsuka et al. 2020) and protecting cochlear sensory cells from acoustic overexposure (Maison and Liberman 2000; Maison et al. 2013; Wolpert et al. 2014; Otsuka et al. 2016). Stronger MOCR suppression facilitates antimasking and noise protective effects, but excessive suppression disrupts the detection of faint signals. Therefore, adaptive MOCR control based on upcoming sound intensity would be a reasonable solution to balance this.

In this study, we performed three experiments to evaluate the effect of expectation with respect to the timing (experiment 1 and 2) and intensity (experiment 3) of an upcoming stimulus on the MOCR. In experiment 1, we assessed the effect of stimulus-driven and exogenous, possibly inflexible and automatic, expectation by applying a preceding visual cue presented at a fixed interval before the MOCR elicitor, such that the physical temporal association between the cue and the MOCR elicitor notified the timing of the upcoming stimulus. In experiment 2, we explored whether the MOCR can be voluntarily controlled in a flexible and dynamic manner, or goal-directed and endogenous manner, by applying a symbolic cue whose appearance indicated the timing of the upcoming MOCR elicitor. In experiment 3, by applying the cued paradigm used in experiment 2, participants were informed about the intensity of the upcoming MOCR elicitor using a visual cue.

## Materials and Methods

### Participants

All participants provided informed consent, and the experiments were approved by the Research Ethics Committee of Chiba University (Chiba, Japan).

A total of 24 volunteers (3 males and 21 females) aged 21–32 years participated in experiment 1, and 12 of them (1 male and 11 females) were subjected to timing-unpredictable conditions. A total of 11 volunteers (2 males and 9 females) aged 21–24 years participated in experiment 2, and 12 of them (3 males and 9 females) participated in experiment 3. Some volunteers were tested in more than one experiment, and in these cases, the order of the experiments was randomized for each participant to minimize the possible confounding effects of learning.

### Equipment

Stimuli were digitally synthesized at a sampling rate of 48 kHz and converted to analog signals using a Roland OCTA-CAPTURE audio interface (16 bits; Roland). Analog signals were amplified by a headphone buffer and presented through Etymotic Research ER-2 earphones (Etymotic) connected to an ER-10B low-noise microphone system (Etymotic). Ear-canal sound pressure was recorded using an Etymotic Research ER-10B low-noise microphone system (Etymotic) inserted into each ear. Prior to the measurements, the outputs from the ER-2 were calibrated using a DB2012 accessory (External Ear Simulator) for the Ear Simulator Type 4257 system (Brüel and Kjær, Nærum, and Denmark). Visual stimuli were displayed on a 10-inch LCD monitor (900 × 600). The viewing distance was ~120 cm, and the display height was ~90 cm. Participants were explicitly instructed to sit up straight, to not move away from or toward the display, and to maintain their gaze on the fixation point at the center of the display.

### Assessment of MOCR Function

MOCR function was noninvasively evaluated through contralateral suppression of OAEs, which are sounds that originate in the cochlea and reflect OHC motility (Kemp 1978). Contralateral suppression of OAEs refers to a reduction in OAE amplitude induced by contralateral acoustic stimulation. This effect is attributed to alterations in OHC motility mediated by the MOCR, which is induced by contralateral acoustic stimulation (Collet et al. 1990).

For measuring OAEs, click trains were presented to the right ear. The clicks had a duration of 100 μs and were presented at a 60-dB peak-equivalent sound pressure level (SPL) and at a rate of 50 times/s. For eliciting the MOCR, the noise was presented to the left ear and band-pass filtered between 100 and 10 000 Hz with a duration of 500 ms, including a 10-ms raised-cosine ramp. It is known that contralateral acoustic stimulation also induces a MEMR. However, the MEMR is generally induced by high-level sounds (>75 dB SPL). In our experiments, the MOCR elicitor was presented at 60-dB SPL. Hence, the OAE suppression observed in our experiment would be dominated by the MOCR.

### Experiment 1: Temporal Expectation Induced by Visual Cue Presentation

Contralateral noise-induced MOCR was compared with and without a visual cue presented immediately before the MOCR elicitor during a timing-predictable and a timing-unpredictable condition. The visual cue consisted of a 10-cm square cross presented on a display. Participants pressed a button once when they heard the sound without visual cue and twice when they heard it with the cue. Participants were instructed to press the button slightly after the noise ended to avoid data contamination with artifacts associated with button presses.

In the timing-predictable condition, the interstimulus interval (ISI) between the visual cue and the MOCR elicitor was fixed across trials (250 ms), such that participants could predict the timing of the MOCR elicitor (Fig. 2*A*). In the timing-unpredictable condition, the ISI changed across trials (randomly chosen between 250, 750, 1250, and 1750 ms), such that participants could not predict the exact timing of the MOCR elicitor (Fig. 2*B*). In both conditions, the MOCR elicitor was randomly presented 30 times for without-cue trials and 120 times for with-cue trials. The order of the trials within the two conditions was randomized across participants. The between-trial interval was randomized and ranged between 2 and 7 s. To avoid foreperiod effects, the comparison of OAE suppression was performed for data generated for the same time interval, that is, 250 ms.

### Experiment 2: Temporal Expectation Induced by Visual Cue Size

We explored whether the MOCR can be voluntarily controlled, so that attention can be flexibly and dynamically shifted based on stimulus-driven temporal expectations.

The timing of the MOCR elicitor presentation was indicated by the size of the visual cue, whereby the appearance of a big (10 cm) or small (5 cm) cross primed the subjects to expect a long (1250 ms) or short (250 ms) ISI, respectively. Participants pressed the button once when hearing the noise without a visual cue and twice when hearing the noise with a visual cue. Participants were instructed to press the button slightly after the noise ended to avoid data contamination with artifacts associated with button presses. Unexpectedly late and unexpectedly early conditions were tested. In the former, one measurement block comprised 30 expectedly late trials (i.e., the cue accurately predicted the 1250-ms interval before the MOCR elicitor), 30 unexpectedly late trials (i.e., the cue predicted a short interval, but the MOCR elicitor appeared after 1250 ms), and 120 expectedly early trials (i.e., the cue accurately predicted the 250-ms interval before the MOCR elicitor). In the unexpectedly early condition, one measurement block comprised 30 expectedly early and 30 unexpectedly early trials (i.e., the cue predicted late intervals, but the MOCR elicitor appeared after 250 ms) and 120 expectedly late trials. The order of trials within the two conditions was randomized across participants. The between-trial onset interval was 2 s.

### Experiment 3: Intensity Expectation Induced by Visual Cue Size

Utilizing a paradigm similar to that employed in experiment 2, we examined whether intensity expectations can modulate the MOCR. The intensity of the MOCR elicitor was indicated by the size of the visual cue, whereby the appearance of a big (10 cm) or small (5 cm) cross primed the subjects to expect a low- or high-intensity sound, respectively. Unexpectedly stronger and unexpectedly weaker conditions were tested. In the former, one measurement block comprised 30 expectedly stronger trials (i.e., the cue accurately predicted a 60-dB SPL sound), 30 unexpectedly stronger trials (i.e., the cue predicted a weak sound, which was instead presented at 60-dB SPL), and 120 expectedly weaker trials (i.e., the cue accurately predicted a weak sound presented at 40-dB SPL). In the unexpectedly weaker condition, one measurement block comprised 30 expectedly weaker trials (i.e., the cue predicted a weak sound presented at 60-dB SPL), 30 unexpectedly weaker trials (i.e., the cue predicted a weak sound, which was instead presented at 60-dB SPL), and 120 expectedly stronger trials (i.e., the cue accurately predicted a strong sound presented at 80-dB SPL). The order of trials within the two conditions was randomized across participants. The between-trial onset interval was 2 s.

### Data Analysis

The recorded signals were band-pass filtered between 1 and 4 kHz to observe the MOCR-related suppression of click-evoked OAEs. Signals were divided into epochs with duration of 2.5 s, starting and ending 0.5 s before and 1 s after the onset of the MOCR elicitor, respectively. The number of epochs was equalized across the trial types. For each of the trials in each condition, 30 out of 120 epochs were randomly selected. For the with-cue trials in the timing-unpredictable condition, 30 epochs with a 250-ms ISI were selected. To maintain an acceptable signal-to-noise ratio, a lower limit of 25 artifact-free epochs per trial and condition was selected. The selected 25 epochs were averaged across trials for each condition, and a time series composed of 125 OAE waveform samples were obtained from the averaged epoch. To smooth the fluctuations included in the time series, 10 adjacent OAE waveform samples were averaged for each time point. The OAE level was calculated as a root mean square (RMS) value for each waveform sample in the 8–18 ms region of the waveform. Finally, an MOCR time course was obtained by subtracting the baseline level from the time series of OAE levels. The baseline level was defined as the average OAE level in the 1-s period before the onset of the first stimulus in a series. The strength of the MOCR for each time course was defined as the mean suppression between 0.25 and 0.75 s after the onset of the preceding sound. We also calculated the RMS for the 0–4 ms region of each waveform sample that was band-pass filtered from 0.1 and 1 kHz (${L}_{0-4\ \mathrm{ms}}$), which is a measurement of MEMR strength. Such early portions of the waveforms reflect the ringing of the click stimulus inside the ear canal, which can be utilized to assess eardrum reflectance and thereby MEMR-induced changes in middle ear transmission (Feeney and Keefe 1999; Schairer et al. 2007).

### Statistical Analysis

In experiments 1 and 3, a paired *t*-test was performed with the cuing and intensity of the MOCR elicitor (with and without the visual cue in experiment 1; expectedly and unexpectedly weaker, expectedly and expectedly stronger in experiment 3). In experiment 2, a repeated-measures analysis of variance (ANOVA) was performed with the timing of the MOCR elicitor (expectedly early, expectedly late, and unexpectedly late) as within-subjects factor. In addition, a repeated-measures ANOVA was also used to assess ${L}_{0-4\ \mathrm{ms}}$. The Ryan–Einot–Gabriel–Welsh F procedure (REGWF) was employed for post-hoc comparisons.

## Results

### Ear Characteristics

**Figure 1:**
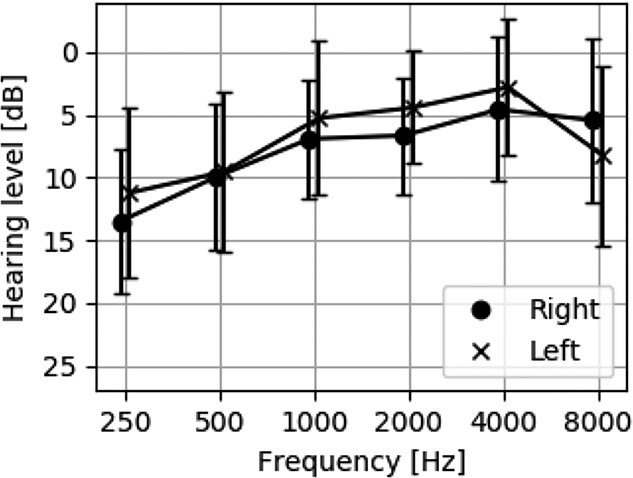
The mean and SD (error bar) of the participants’ hearing levels.

The ears of all participants had normal pure-tone audiometric thresholds (hearing loss < 20 dB) ranging from 0.5 to 8 kHz. All ears showed normal tympanogram results; the peak-compensated static compliance was 0.3–2.0 mL, and peak pressure ranged between −100 and +50 daPa. The mean peak-compensated static compliance was 0.84 (standard deviation [SD] = 0.39) for the right ear and 0.82 (SD = 0.39) for the left ear. The mean peak pressure was −9.7 (SD = 18.6) and −10.6 (SD = 19.3) for the right and left ear, respectively. The audiometry results are shown in Figure 1.

### The Effects of Visual Cue Presentation on Temporal Expectation and the MOCR

**Figure 2:**
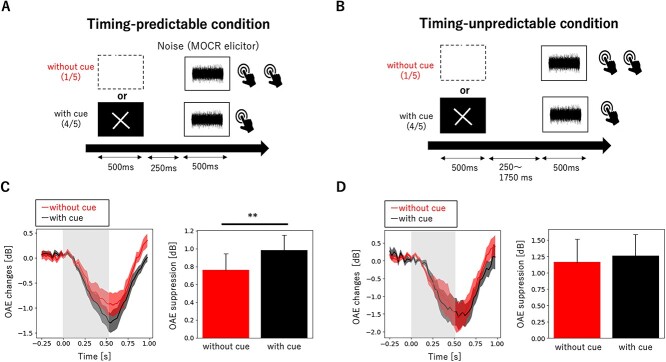
Visual signal-induced temporal expectation modulates the MOCR, but only when the visual cue predicts stimulus onset timing*.* (*A*, *B*) Schematic representation of the task. Participants maintained their gaze on a fixation point. A 60-dB-SPL noise was contralaterally presented to elicit the MOCR and preceded by a visual cue. (*A*) In the timing-predictable condition, the ISI between the visual cue and the noise was constant at 250 ms. (*B*) In the timing-unpredictable condition, the ISI was randomly chosen between 250, 750, 1250, and 1750 ms, such that the subjects could not predict the exact timing of noise presentation. In both conditions, in one of five trials, the noise was presented without a preceding visual cue. The interval between the noise end and the onset of the next visual stimulus varied from 2 to 7 s for every trial. Participants pressed a button once when they heard the noise without visual cue and twice when they heard it with it. Participants were instructed to press the button slightly after the noise ended to avoid data contamination with artifacts associated with button presses. (*C*, *D*) Grand average of the time course of OAE suppression induced by the MOCR elicitor (left panels) and maximum OAE suppression with and without a preceding cue (right panels). (*C*) In the time-predictable condition, maximum OAE suppression was significantly stronger in trials with a visual cue compared with those without a preceding cue. (*D*) In the timing-unpredictable condition, there was no difference in OAE suppression between conditions with and without visual cue. The comparison between the two conditions was performed for data across the same time interval, that is, 250 ms. The light gray color indicates the duration of the MOCR elicitor. The light-colored area depicts the standard error. Error bars represent the standard error of the mean. ^**^P < 0.01 (paired *t*-test).

The participants enrolled in experiment 1 had a mean age of 23.3 years (SD = 3.7). In the timing-predictable condition, visual cue presentation led to a stronger OAE suppression compared with the without-cue condition (*T* = −3.0, *P* = 0.0069; Fig. 2*C*). Mean OAE suppression with and without the visual cue was 0.99 dB (SD = 0.81) and 0.76 dB (SD = 0.90), respectively. ${L}_{0-4\ \mathrm{ms}}$ with and without the visual cue was 0.46 dB (SD = 0.90) and 0.51 dB (SD = 1.85), which were not significantly different from zero (*T* = 2.45, *P* = 0.022; *T* = 1.33, *P* = 0.19) and did not statistically differ between each other (*T* = 0.15, *P* = 0.88).

In contrast, there was no difference in OAE suppression with or without the visual cue in the timing-unpredictable condition (*T* = −0.58, *P* = 0.57; Fig. 2*D*). Mean OAE suppression with and without the visual cue was 1.26 dB (SD = 1.12) and 1.16 dB (SD = 1.23), respectively. ${L}_{0-4\ \mathrm{m}s}$ with and without the visual cue was 0.016 dB (SD = 0.062) and 0.022 dB (SD = 0.062), which were not significantly different from zero (*T* = 0.83, *P* = 0.42; *T* = 1.17, *P* = 0.27) and did not statistically differ between each other (*T* = 0.94 *P* = 0.37).

### The Effects of Visual Cue Size on Temporal Expectation and the MOCR

**Figure 3:**
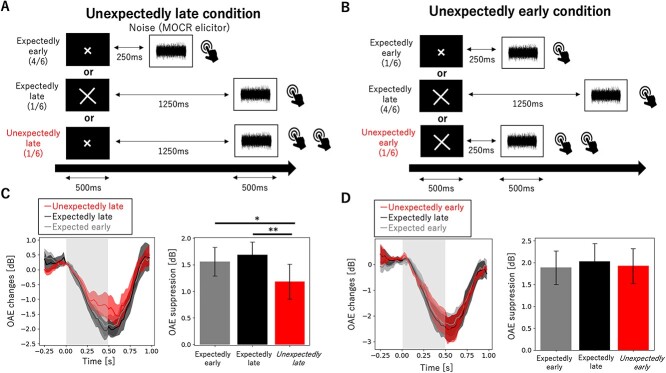
Unexpectedly late sounds induce a weak MOCR, while unexpectedly early sounds induce a strong MOCR that is comparable to that elicited by sounds appearing at the expected moment. (*A*, *B*) Schematic representation of the task. Participants maintained their gaze on a fixation point in the center of the screen and were informed that a brief visual cue (either a small or big cross) indicating the ISI length (250 or 1250 ms) would follow. The trial rate for each combination is reported in parentheses. Participants pressed the button once when hearing the noise appearing at an expected timing and twice when hearing the noise appearing at an unexpected timing. Participants were instructed to press the button slightly after the noise ended to avoid data contamination with artifacts associated with button presses. (*A*) In the unexpectedly late condition, the noise appeared later than expected, once every six trials. (*B*) In the unexpectedly early condition, the noise appeared earlier than expected, once every six trials. (*C*, *D*) Grand average of the time course of OAE suppression induced by the MOCR elicitor (left panels) and maximum OAE suppression for trials with and without the preceding cue (right panels). (*C*) Sounds appearing unexpectedly late induced a weak MOCR, (*D*) while those that appeared unexpectedly early induced a strong MOCR, which was comparable to that appearing at the expected moment. Error bars represent the standard error of the mean. ^*^P < 0.05, ^**^P < 0.01 (corrected for multiple comparisons with the REGWF procedure).

The participants enrolled in experiment 2 had a mean age of 21.3 years (SD = 2.4). The results of experiment 2 showed that sounds occurring unexpectedly later induced a weaker MOCR than those occurring expectedly later and those occurring earlier than expected induced a strong MOCR that was comparable to that elicited by expectedly early stimuli. Mean OAE suppression in the expectedly early, expectedly late, and unexpectedly late condition was 1.56 dB (SD = 0.90), 1.68 dB (SD = 0.80), and 1.18 dB (SD = 1.09), respectively. In addition, the timing of the MOCR elicitor had a significant effect on OAE suppression (*F*_2, 20_ = 5.5, *P* = 0.012; Fig. 3*C*). Post-hoc comparison showed that unexpectedly late eliciting sound occurrence induced weaker OAE suppression compared with expectedly late (*T* = 3.2, *P* = 0.0046 < nominal level of *P* < 0.01; Fig. 3*C*) or early (*T* = 2.4, *P* = 0.027 < nominal level of *P* < 0.05; Fig. 3*C*) sound onset. Mean ${L}_{0-4\ \mathrm{ms}}$ changes in the expectedly early, expectedly late, and unexpectedly late conditions were 0.063 dB (SD = 0.19), 0.14 dB (SD = 0.21), and −0.081 dB (SD = 0.39), which were not significantly different from zero (*T* = 1.02, *P* = 0.33; *T* = 2.13, *P* = 0.059; *T* = −0.67, *P* = 0.52, respectively). In addition, the timing of the MOCR elicitor had no effect on ${L}_{0-4\ \mathrm{ms}}$(*F*_2, 20_ = 1.80, *P* = 0.19).

**Figure 4:**
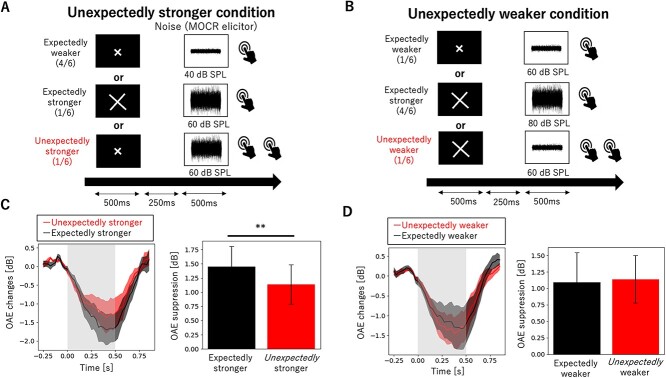
An unexpectedly stronger stimulus induces a weaker MOCR than a stimulus with an expectedly stronger intensity, whereas unexpectedly weaker sounds induce a weak MOCR, comparable with that appearing at an expectedly weaker intensity. (*A*, *B*) Schematic representation of the task. Participants maintained their gaze on a fixation point at the center of the screen and were told that a brief visual cue (either a small or big cross) indicated the intensity of the upcoming stimulus (40- or 60-dB SPL in the unexpectedly stronger condition, 60- or 80-dB SPL in the unexpectedly weaker condition). The ISI between cue and noise was fixed at 250 ms. Participants pressed a button once when they heard the noise appearing at an expected intensity, and twice when they heard the noise appearing at an unexpected intensity. Participants were instructed to press the button slightly after the noise ended to avoid data contamination with artifacts associated with button presses. (*A*) To examine the effect of an invalid cue on the MOCR, in the unexpectedly stronger condition, a stronger intensity noise appeared after the small cross once every six trials. (*B*) In contrast, in the unexpectedly weaker condition, a weaker noise appeared after the large cross once every six trials. The trial rates for each combination are reported in parentheses. (*C*, *D*) Grand average of the time course of OAE suppression induced by the MOCR elicitor (left panels) and maximum OAE suppression for trials with and without a preceding cue (right panels). (*C*) The MOCR induced by the unexpectedly stronger condition was weaker than that induced by the expectedly stronger condition, whereas (*D*) stimuli weaker than expected elicited an MOCR comparable to that elicited by expectedly weaker stimuli. Error bars represent the standard error of the mean. ^**^P < 0.01 (corrected for multiple comparisons with the REGWF procedure).

In contrast, unexpectedly early eliciting sound occurrence induced an OAE suppression that was comparable to that elicited by expectedly late and early sounds (*F*_2, 20_ = 0.12, *P* = 0.89; Fig. 3*D*). Mean OAE suppression in the expectedly early, expectedly late, and unexpectedly late conditions was 1.89 dB (SD = 1.27), 2.03 dB (SD = 1.35), and 1.93 dB (SD = 1.32), respectively. Mean ${L}_{0-4\ \mathrm{ms}}$ changes in the expectedly early, expectedly late, and unexpectedly late conditions were 0.072 dB (SD = 0.18), −0.022 dB (SD = 0.10), and −0.053 dB (SD = 0.17), which were not significantly different from zero (*T* = 1.29, *P* = 0.23; *T* = −0.67, *P* = 0.52; *T* = −0.97, *P* = 0.35, respectively). In addition, the timing of the MOCR elicitor had no effect on ${L}_{0-4\ \mathrm{ms}}$(*F*_2, 20_ = 1.71, *P* = 0.21).

### The Effects of Visual Cue Size on Intensity Expectation and the MOCR

The participants enrolled in experiment 3 had a mean age of 22.7 years (SD = 1.7). An unexpectedly stronger eliciting sound induced a weaker OAE suppression than that induced by an expectedly stronger eliciting sound (*T* = 3.3, *P* = 0.0074; Fig. 4*C*). Mean OAE suppression in the expectedly stronger and unexpectedly stronger conditions was 1.45 dB (SD = 1.24) and 1.14 dB (SD = 1.20), respectively. ${L}_{0-4\ \mathrm{ms}}$ in the expectedly stronger and unexpectedly stronger conditions was 0.029 dB (SD = 0.22) and 0.12 dB (SD = 0.31), which were not significantly different from zero (*T* = 0.45 *P* = 0.66; *T* = 1.22, *P* = 0.25, respectively) and did not statistically differ between each other (*T* = −1.43 *P* = 0.18). In contrast, an unexpectedly weaker eliciting sound induced an OAE suppression that was comparable to that induced by an expectedly weaker eliciting sound (*T* = 0.29, *P* = 0.77; Fig. 4*D*). Mean OAE suppression in the expectedly stronger and unexpectedly stronger conditions was 1.09 dB (SD = 1.56) and 1.14 dB (SD = 1.24), respectively. ${L}_{0-4\ \mathrm{ms}}$ in the expectedly stronger and unexpectedly stronger conditions was 0.056 dB (SD = 0.23) and −0.0017 dB (SD = 0.19), which were not significantly different from zero (*T* = 0.80 *P* = 0.44; *T* = −0.029 *P* = 0.98, respectively) and did not statistically differ between each other (*T* = 0.53 *P* = 0.61).

## Discussion

We found that the MOCR was enhanced by a warning signal presented when the ISI was fixed but not when the interval changed across trials. In addition, a stronger MOCR was observed when the learned timing expectation matched with the timing of the sound but remained unvaried when these two factors did not match. These findings indicate that the MOCR can be voluntarily controlled in a goal-oriented and not only a stimulus-driven manner. By applying a similarly cued paradigm, we further showed that the MOCR is enhanced by the expectation of a stronger, but not of weaker, sound intensity. In contrast, the ringing inside the ear canal (${L}_{0-4\ \mathrm{ms}}$), which would reflect middle ear transmission and thereby MEMR, did not differ. These findings indicate that expectations relevant to the timing and intensity of upcoming sounds can modulate the MOCR, but not the MEMR, under flexible and preparatory control, thereby influencing the sensory transduction phase.

Top-down circuits are ubiquitous in the central nervous system (Elgueda and Delano 2020). The auditory cortex receives and is modulated by descending projections from other cortical areas, such as the frontal and nonauditory cortex, which create an attentional processing loop (Winer and Lee 2007). The auditory descending pathway originates in the auditory cortex and projects to the subcortical nucleus, reaching the cochlea through MOC fibers (Winer 2005; Terreros and Delano 2015). These connections form a feedback loop that initiates and reinforces altered neural sound representations along the central auditory pathway (Suga and Ma 2003). Focal electrical stimulation in the auditory cortex evokes highly specific changes in the frequency, intensity, location, and duration of potentials in subcortical neurons (reviewed in King and Bajo 2013; Schofield and Beebe 2019; Suga and Ma 2003). The cortically driven modulation plays a role in perceptual learning (Bajo et al. 2010) and is hypothesized to mediate attentional modulation of auditory processing (Xiao and Suga 2002).

Concomitantly, previous literature has reported the positive effect of the MOCR on attention, despite some negative results (de Boer and Thornton 2007; Beim et al. 2018); studies measuring OAE-based MOCR in humans and experimental animals have shown that orienting attention to a specific laterality (Froehlich et al. 1993), auditory target (Smith et al. 2012), frequency (Maison et al. 2001), working memory task (Marcenaro et al. 2021), and visual task (Delano et al. 2007; Wittekindt et al. 2014) modulates the MOCR. Auditory training also enhances the MOCR, leading to speech-in-noise perception facilitation (de Boer and Thornton 2008).

Anderson and Malmierca (2013) further showed that inactivation of the auditory cortex modulates stimulus-specific adaptation (SSA) of cells in subcortical areas (Anderson and Malmierca 2013). As SSA is a plausible mechanism underlying predictive coding (Baldeweg 2006; Winkler et al. 2009; Bendixen et al. 2012; Malmierca et al. 2015), the descending pathway may be related to forming or facilitating predictive processing at subcortical levels (Malmierca et al. 2015). In line with these animal studies, Riecke et al. (2020) found that the predictability of the frequency of upcoming tones alters OAE amplitude in a fashion that depends on the behavioral relevance of the tone sequences (Riecke et al. 2020). In addition, the authors reported a significant correlation between the increase in OAE amplitude and cortical auditory event-related potential in the case of predictability. This correlation provides evidence that auditory predictions concerning the frequency of a sound exert a top-down effect on the sensory processing of the auditory periphery via the corticofugal pathway. However, the present study is the first to show evidence that the descending pathway conveys an expectation about timing and intensity of an upcoming sound to the first stage of auditory processing, that is, the sensory transduction phase.

One may think that the participants just detected the visual cue but did not form any expectation concerning the timing of sound occurrence. However, in experiment 1, we found that the warning cue enhanced MOCR in the case that the ISI remains unchanged between the cue and the MOCR elicitor, which suggests that MOCR enhancement reflects increased preparatory processes that become engaged by timing predictability. However, this datum does not suggest enhanced general attention readiness induced by the preceding visual cue. In addition, the lack of significant MOCR changes in the timing-unpredictable condition also implies that the number of button pressings did not influence the results. In experiment 2, where the visual stimulus appeared both in the expected and unexpected condition, the differences in the MOCR can be attributed to the temporal predictability of the elicitor. Therefore, the current study showed that stimuli that occur at expected times induce a stronger MOCR.

Similarly, previous studies have reported increased cortical responses to stimuli appearing at the expected moment, presumably due to expectation-mediated orienting of attention to the event (Ghose and Maunsell 2002; Lange et al. 2003; Doherty et al. 2005; Praamstra et al. 2006; Jaramillo and Zador 2011; Auksztulewicz et al. 2019). The timing-specific increase in neural excitability, or temporal attention, is plausibly underpinned by the entrainment of low-frequency cortical oscillations (<10 Hz, including delta, theta, and low alpha bands) by periodic, thereby temporally predictable, stimulation (Lakatos et al. 2008; Schroeder and Lakatos 2009; Arnal and Giraud 2012; Auksztulewicz et al. 2019), and aperiodic stimulation when the timing of the stimulus occurrence is predictable (Morillon et al. 2016; Breska and Deouell 2017; Rimmele et al. 2018). Increased cortical neural entrainment associated with temporal attention could modulate the activity of MOC neurons via the corticofugal pathway, which could thus underlie the enhanced OAE suppression observed in our study. In line with this hypothesis, Dragicevic et al. (2019) provided evidence of the interaction between OAEs and low-frequency cortical oscillations during selective attention, which supports that attentional processing at the cortical level can modulate the OHC gain via the corticofugal pathways. In contrast, temporal predictability has also been reported to suppress cortical and brainstem potentials (Lange 2009; Costa-Faidella et al. 2011; Todorovic et al. 2011; Gorina-Careta et al. 2016), which could be evidence of the predictive coding hypothesis, which posits that the neural responses to expected stimuli should be suppressed (Friston 2005). Enhanced MOCR associated with temporal predictability, which leads to increased suppression of the cochlear response, can be understood as a part of a prediction-based inhibition network underling the predictive coding framework.

The descending pathway from the auditory cortex is not the only pathway that can modulate the MOCR. Studies on animal models have demonstrated the presence of corticofugal projections from the frontal cortex to the inferior colliculus (Olthof et al. 2019; Elgueda and Delano 2020), which could be an alternative circuit to the top-down MOC efferent pathway. In addition, the enhancement of the MOCR can be explained by the increased firing rate of auditory nerves via the lateral olivocochlear (LOC) fibers, which innervate auditory nerves (Guinan 2006). However, the attentional modulation possibly operated by the LOC system has not been examined.

Previous psychological data have shown that the temporal orienting effect on target detection is only significant for invalidly cued targets that appear earlier than expected (Coull and Nobre 1998; Miniussi et al. 1999; Griffin et al. 2001, 2002). A similar dependence of responses on the foreperiod interval length has been observed in multiple brain areas, as revealed by event-related potentials, which reflect the decision-making state or memory trace originating in the prefrontal area (Miniussi et al. 1999; Griffin et al. 2001, 2001), and by single-cell recordings in the auditory cortex (Jaramillo and Zador 2011). A possible explanation for the previous results is that invalidly cued targets appearing later than expected provide enough time for attention to be re-oriented to the later interval as a result of increasing conditional probabilities over time.

However, we found that, when the elicitor is presented earlier than expected, the degree of the MOCR is comparable to that occurring at the expected moment. This result implies that MOCR enhancement starts immediately after cue presentation and lasts until the expected time, disappearing afterward. This discrepancy in the dependance of the response on the length of foreperiod interval suggests that the MOCR can be voluntarily controlled in a flexible or dynamic manner but is not controlled by the re-orienting effect according to conditional probabilities. Although the neural substrate that modulates MOCR responses according to temporal expectation is likely to correspond to the top-down control of the auditory cortex via corticofugal projections to the subcortical MOCR circuits (Terreros and Delano 2015), its principal operation would differ from that in cortical areas and might be aimed at fast updating subsequent expectations after the expected moment. Alternatively, the weaker MOCR observed when the learned expectation and timing match might be attributed to the release of the learned expectation from working memory. Marcenaro et al. (2021) found that larger acoustic suppression of distortion-product OAEs arise during a visual working memory task than during control conditions, in which the same stimuli as those of the working memory task were presented, but no task was performed (Marcenaro et al. 2021). In a cuing task, like in a working memory task, participants are forced to retain what a cue indicates until the expected timing of event occurrence, which would enhance the MOCR-related OAE suppression. After the expected event occurrence, listeners might release the memory, and therefore, the enhancement of OAE suppression would disappear.

With respect to optimization, our result that the MOCR is enhanced by expectation of stronger, but not weaker, intensity sounds is reasonable. As mentioned above, the MOCR inhibits OHC motility and protects the sensory system from acoustic overexposure (Maison and Liberman 2000; Otsuka et al. 2016). The suppression induced by the MOCR also improves the detection of signals in noise by preventing the adaption of auditory nerves to the noise and maintaining their responsiveness to upcoming targets (Kawase and Liberman 1993; Kumar and Vanaja 2004; Otsuka et al. 2020). Stronger suppression facilitates noise protection and antimasking effects, but too strong suppression disrupts the detection of faint signals. In this sense, the enhancement of the MOCR, that is, a stronger suppression only for stronger sounds, could be a reasonable solution to find balance in the trade-off.

## References

[ref1] Amit R, Abeles D, Carrasco M, Yuval-Greenberg S. 2019. Oculomotor inhibition reflects temporal expectations. NeuroImage. 184:279–292. 30223059 10.1016/j.neuroimage.2018.09.026

[ref2] Anderson LA, Malmierca MS. 2013. The effect of auditory cortex deactivation on stimulus-specific adaptation in the inferior colliculus of the rat. Eur J Neurosci. 37:52–62. 23121128 10.1111/ejn.12018

[ref3] Arnal LH, Giraud AL. 2012. Cortical oscillations and sensory predictions. Trends Cogn Sci. 16:390–398. 22682813 10.1016/j.tics.2012.05.003

[ref4] Auksztulewicz R, Myers NE, Schnupp JW, Nobre AC. 2019. Rhythmic temporal expectation boosts neural activity by increasing neural gain. J Neurosci. 39:9806–9817. 31662425 10.1523/JNEUROSCI.0925-19.2019 PMC6891052

[ref5] Backus BC, Guinan JJ. 2006. Time-course of the human medial olivocochlear reflex. J Acoust Soc Am. 119:2889–2904. 16708947 10.1121/1.2169918

[ref6] Bajo VM, Nodal FR, Moore DR, King AJ. 2010. The descending corticocollicular pathway mediates learning-induced auditory plasticity. Nat Neurosci. 13:253–260. 20037578 10.1038/nn.2466 PMC3634157

[ref7] Baldeweg T . 2006. Repetition effects to sounds: evidence for predictive coding in the auditory system. Trends Cogn Sci. 10:93–94. 16460994 10.1016/j.tics.2006.01.010

[ref8] Barnes R, Jones MR. 2000. Expectancy, attention, and time. Cogn Psychol. 41:254–311. 11032658 10.1006/cogp.2000.0738

[ref9] Beim JA, Oxenham AJ, Wojtczak M. 2018. Examining replicability of an otoacoustic measure of cochlear function during selective attention. J Acoust Soc Am. 144:2882–2895. 30522315 10.1121/1.5079311 PMC6246073

[ref10] Bendixen A, SanMiguel I, Schröger E. 2012. Early electrophysiological indicators for predictive processing in audition: a review. Int J Psychophysiol. 83:120–131. 21867734 10.1016/j.ijpsycho.2011.08.003

[ref11] Betta E, Turatto M. 2006. Are you ready? I can tell by looking at your microsaccades. Neuroreport. 17:1001–1004. 16791092 10.1097/01.wnr.0000223392.82198.6d

[ref12] Binda P, Gamlin PD. 2017. Renewed attention on the pupil light reflex. Trends Neurosci. 40:455–457. 28693846 10.1016/j.tins.2017.06.007 PMC5562352

[ref15] Borg E, Zakrisson JE. 1975. The activity of the stapedius muscle in man during vocalization. Acta Otolaryngol. 79:325–333. 1155042 10.3109/00016487509124694

[ref16] Breska A, Deouell LY. 2017. Neural mechanisms of rhythm-based temporal prediction: delta phase-locking reflects temporal predictability but not rhythmic entrainment. PLoS Biol. 15:e2001665. 28187128 10.1371/journal.pbio.2001665 PMC5302287

[ref17] Collet L, Kemp DT, Veuillet E, Duclaux R, Moulin A, Morgon A. 1990. Effect of contralateral auditory stimuli on active cochlear micro-mechanical properties in human subjects. Hear Res. 43:251–261. 2312416 10.1016/0378-5955(90)90232-e

[ref18] Correa Á, Lupiáñez J, Tudela P. 2005. Attentional preparation based on temporal expectancy modulates processing at the perceptual level. Psychon Bull Rev. 12:328–334. 16082814 10.3758/bf03196380

[ref19] Correa Á, Lupiáñez J, Madrid E, Tudela P. 2006. Temporal attention enhances early visual processing: a review and new evidence from event-related potentials. Brain Res. 1076:116–128. 16516173 10.1016/j.brainres.2005.11.074

[ref20] Costa-Faidella J, Baldeweg T, Grimm S, Escera C. 2011. Interactions between “what” and “when” in the auditory system: temporal predictability enhances repetition suppression. J Neurosci. 31:18590–18597. 22171057 10.1523/JNEUROSCI.2599-11.2011 PMC6623902

[ref21] Coull JT, Nobre AC. 1998. Where and when to pay attention: the neural systems for directing attention to spatial locations and to time intervals as revealed by both PET and fMRI. J Neurosci. 18:7426–7435. 9736662 10.1523/JNEUROSCI.18-18-07426.1998 PMC6793260

[ref22] Coull JT, Frith CD, Büchel C, Nobre AC. 2000. Orienting attention in time: behavioural and neuroanatomical distinction between exogenous and endogenous shifts. Neuropsychologia. 38:808–819. 10689056 10.1016/s0028-3932(99)00132-3

[ref23] Coull JT, Nazarian B, Vidal F. 2008. Timing, storage, and comparison of stimulus duration engage discrete anatomical components of a perceptual timing network. J Cogn Neurosci. 20:2185–2197. 18457512 10.1162/jocn.2008.20153

[ref24] Cravo AM, Rohenkohl G, Wyart V, Nobre AC. 2013. Temporal expectation enhances contrast sensitivity by phase entrainment of low-frequency oscillations in visual cortex. J Neurosci. 33:4002–4010. 23447609 10.1523/JNEUROSCI.4675-12.2013 PMC3638366

[ref25] Cui X, Stetson C, Montague PR, Eagleman DM. 2009. Ready…go: amplitude of the fMRI signal encodes expectation of cue arrival time. PLoS Biol. 7:e1000167. 19652698 10.1371/journal.pbio.1000167 PMC2711330

[ref26] Dankner Y, Shalev L, Carrasco M, Yuval-Greenberg S. 2017. Prestimulus inhibition of saccades in adults with and without attention-deficit/hyperactivity disorder as an index of temporal expectations. Psychol Sci. 28:835–850. 28520552 10.1177/0956797617694863

[ref13] de Boer J, Thornton ARD. 2007. Effect of subject task on contralateral suppression of click evoked otoacoustic emissions. Hear Res. 233:117–123. 17910996 10.1016/j.heares.2007.08.002

[ref14] de Boer J, Thornton ARD. 2008. Neural correlates of perceptual learning in the auditory brainstem: efferent activity predicts and reflects improvement at a speech-in-noise discrimination task. J Neurosci. 28:4929–4937. 18463246 10.1523/JNEUROSCI.0902-08.2008 PMC6670751

[ref27] Delano PH, Elgueda D, Hamame CM, Robles L. 2007. Selective attention to visual stimuli reduces cochlear sensitivity in chinchillas. J Neurosci. 27:4146–4153. 17428992 10.1523/JNEUROSCI.3702-06.2007 PMC6672531

[ref28] Doherty JR, Rao A, Mesulam MM, Nobre AC. 2005. Synergistic effect of combined temporal and spatial expectations on visual attention. J Neurosci. 25:8259–8266. 16148233 10.1523/JNEUROSCI.1821-05.2005 PMC6725546

[ref29] Dragicevic CD, Aedo C, León A, Bowen M, Jara N, Terreros G, Robles L, Delano PH. 2015. The olivocochlear reflex strength and cochlear sensitivity are independently modulated by auditory cortex microstimulation. JARO. 16:223–240. 25663383 10.1007/s10162-015-0509-9 PMC4368653

[ref29a] Dragicevic CD, Marcenaro B, Navarrete M, Robles L, Delano PH. 2019. Oscillatory infrasonic modulation of cochlear amplifier by selective attention. Plos One. 14:e0208939. 30615632 10.1371/journal.pone.0208939 PMC6322828

[ref29b] Ebitz RB, Moore T. 2017. Selective modulation of the pupil light reflex by microstimulation of prefrontal cortex. J Neurosci. 37:5008–5018. 28432136 10.1523/JNEUROSCI.2433-16.2017 PMC6596477

[ref30] Elgueda D, Delano PH. 2020. Corticofugal modulation of audition. Curr Opin Physio. 18:73–78.

[ref31] Feeney MP, Keefe DH. 1999. Acoustic reflex detection using wide-band acoustic reflectance, admittance, and power measurements. J Speech Lang Hear Res. 42:1029–1041. 10515503 10.1044/jslhr.4205.1029

[ref32] Friston K . 2005. A theory of cortical responses. Philos Trans of T Soc Lond B, Biol Sci. 360:815–836. 10.1098/rstb.2005.1622 PMC1569488 15937014

[ref33] Froehlich P, Collet L, Morgon A. 1993. Transiently evoked otoacoustic emission amplitudes change with changes of directed attention. Physiol Behav. 53:679–682. 8511172 10.1016/0031-9384(93)90173-d

[ref34] Ghose GM, Maunsell JHR. 2002. Attentional modulation in visual cortex depends on task timing. Nature. 419:616–620. 12374979 10.1038/nature01057

[ref35] Gorina-Careta N, Zarnowiec K, Costa-Faidella J, Escera C. 2016. Timing predictability enhances regularity encoding in the human subcortical auditory pathway. Sci Rep. 6:1–9. 27853313 10.1038/srep37405 PMC5112601

[ref36] Griffin IC, Miniussi C, Nobre AC. 2001. Orienting attention in time. Front Biosci. 6:660–671. 10.2741/griffin 11282565

[ref37] Griffin IC, Miniussi C, Nobre AC. 2002. Multiple mechanisms of selective attention: differential modulation of stimulus processing by attention to space or time. Neuropsychologia. 40:2325–2340. 12417462 10.1016/s0028-3932(02)00087-8

[ref38] Guinan JJ . 2006. Olivocochlear efferents: anatomy, physiology, function, and the measurement of efferent effects in humans. Ear Hear. 27:589–607. 17086072 10.1097/01.aud.0000240507.83072.e7

[ref39] Hackley SA, Schankin A, Wohlschlaeger A, Wascher E. 2007. Localization of temporal preparation effects via trisected reaction time. Psychophysiology. 44:334–338. 17343715 10.1111/j.1469-8986.2007.00500.x

[ref40] Jaramillo S, Zador AM. 2011. The auditory cortex mediates the perceptual effects of acoustic temporal expectation. Nat Neurosci. 14:246–251. 21170056 10.1038/nn.2688 PMC3152437

[ref41] Kawase T, Liberman MC. 1993. Antimasking effects of the olivocochlear reflex. I. Enhancement of compound action potentials to masked tones. J Neurophysiol. 70:2519–2532. 8120596 10.1152/jn.1993.70.6.2519

[ref42] Kemp DT . 1978. Stimulated acoustic emissions from within the human auditory system. J Acoust Soc Am. 64:1386–1391. 744838 10.1121/1.382104

[ref43] King AJ, Bajo VM. 2013. Cortical modulation of auditory processing in the midbrain. Front Neural Circuits. 6:114. 23316140 10.3389/fncir.2012.00114 PMC3539853

[ref44] Kumar UA, Vanaja CS. 2004. Functioning of olivocochlear bundle and speech perception in noise. Ear Hear. 25:142–146. 15064659 10.1097/01.aud.0000120363.56591.e6

[ref45] Lakatos P, Karmos G, Mehta AD, Ulbert I, Schroeder CE. 2008. Entrainment of neuronal oscillations as a mechanism of attentional selection. Science. 320:110–113. 18388295 10.1126/science.1154735

[ref46] Lange K . 2009. Brain correlates of early auditory processing are attenuated by expectations for time and pitch. Brain Cogn. 69:127–137. 18644669 10.1016/j.bandc.2008.06.004

[ref48] Lange K, Rösler F, Röder B. 2003. Early processing stages are modulated when auditory stimuli are presented at an attended moment in time: an event-related potential study. Psychophysiology. 40:806–817. 14696734 10.1111/1469-8986.00081

[ref49] Liberman MC, Guinan JJ. 1998. Feedback control of the auditory periphery: anti-masking effects of middle ear muscles vs. olivocochlear efferents. J Commun Disord. 31:471–483. 9836136 10.1016/s0021-9924(98)00019-7

[ref50] Los SA, Heslenfeld DJ. 2005. Intentional and unintentional contributions to nonspecific preparation: electrophysiological evidence. J Exp Psychol. 134:52–72. 10.1037/0096-3445.134.1.52 15702963

[ref51] Maison SF, Liberman MC. 2000. Predicting vulnerability to acoustic injury with a noninvasive assay of olivocochlear reflex strength. J Neurosci. 20:4701–4707. 10844039 10.1523/JNEUROSCI.20-12-04701.2000 PMC6772446

[ref52] Maison S, Micheyl C, Collet L. 2001. Influence of focused auditory attention on cochlear activity in humans. Psychophysiology. 38:35–40. 11321619

[ref53] Maison SF, Usubuchi H, Liberman MC. 2013. Efferent feedback minimizes cochlear neuropathy from moderate noise exposure. J Neurosci. 33:5542–5552. 23536069 10.1523/JNEUROSCI.5027-12.2013 PMC3640841

[ref54] Malmierca MS, Anderson LA, Antunes FM. 2015. The cortical modulation of stimulus-specific adaptation in the auditory midbrain and thalamus: a potential neuronal correlate for predictive coding. Front Syst Neurosci. 9:19. 25805974 10.3389/fnsys.2015.00019 PMC4353371

[ref55] Marcenaro B, Leiva A, Dragicevic C, López V, Delano PH. 2021. The medial olivocochlear reflex strength is modulated during a visual working memory task. J Neurophysiol. 125:2309–2321. 33978484 10.1152/jn.00032.2020

[ref56] Mathôt S, van der Linden L, Grainger J, Vitu F. 2015. The pupillary light response reflects eye-movement preparation. J Exp Psychol Hum Percept Perform. 41:28–35. 25621584 10.1037/a0038653

[ref57] Miniussi C, Wilding LE, Coull TJ, Nobre CA. 1999. Orienting attention in time: modulation of brain potentials. Brain. 122:1507–1518. 10430834 10.1093/brain/122.8.1507

[ref58] Morillon B, Schroeder CE, Wyart V, Arnal LH. 2016. Temporal prediction in lieu of periodic stimulation. J Neurosci. 36:2342–2347. 26911682 10.1523/JNEUROSCI.0836-15.2016 PMC4860448

[ref59] Mukerji S, Windsor AM, Lee DJ. 2010. Auditory brainstem circuits that mediate the middle ear muscle reflex. Trends Amplif. 14:170–191. 20870664 10.1177/1084713810381771 PMC3624626

[ref60] Niemi P, Lehtonen E. 1982. Foreperiod and visual stimulus intensity: a reappraisal. Acta Psychol. 50:73–82. 10.1016/0001-6918(82)90052-x 7054926

[ref61] Nobre AC, van Ede F. 2018. Anticipated moments: temporal structure in attention. Nat Rev Neurosci. 19:34–48. 29213134 10.1038/nrn.2017.141

[ref62] Olthof BMJ, Gartside SE, Rees A. 2019. Puncta of neuronal nitric oxide synthase (nNOS) mediate NMDA receptor signaling in the auditory midbrain. J Neurosci. 39:876–887. 30530507 10.1523/JNEUROSCI.1918-18.2018 PMC6382984

[ref63] Otsuka S, Tsuzaki M, Sonoda J, Tanaka S, Furukawa S. 2016. A role of medial olivocochlear reflex as a protection mechanism from noise-induced hearing loss revealed in short-practicing violinists. PLoS One. 11:e0146751. 26745634 10.1371/journal.pone.0146751 PMC4706422

[ref64] Otsuka S, Nakagawa S, Furukawa S. 2020. Relationship between characteristics of medial olivocochlear reflex and speech-in-noise-reception performance. Acoust Sci and Technol. 41:404–407.

[ref65] Palmer SE, Marre O, Berry MJ, Bialek W. 2015. Predictive information in a sensory population. PNAS. 112:6908–6913. 26038544 10.1073/pnas.1506855112 PMC4460449

[ref67] Praamstra P, Kourtis D, Kwok HF, Oostenveld R. 2006. Neurophysiology of implicit timing in serial choice reaction-time performance. J Neurosci. 26:5448–5455. 16707797 10.1523/JNEUROSCI.0440-06.2006 PMC6675318

[ref68] Rao RPN, Ballard DH. 1999. Predictive coding in the visual cortex: a functional interpretation of some extra-classical receptive-field effects. Nat Neurosci. 2:79–87. 10195184 10.1038/4580

[ref69] Riecke L, Marianu IA, De Martino F. 2020. Effect of auditory predictability on the human peripheral auditory system. Front Neurosci. 14:362. 32351361 10.3389/fnins.2020.00362 PMC7174672

[ref70] Rimmele JM, Morillon B, Poeppel D, Arnal LH. 2018. Proactive sensing of periodic and aperiodic auditory patterns. Trends Cogn Sci. 22:870–882. 30266147 10.1016/j.tics.2018.08.003

[ref71] Rohenkohl G, Cravo AM, Wyart V, Nobre AC. 2012. Temporal expectation improves the quality of sensory information. J Neurosci. 32:8424–8428. 22699922 10.1523/JNEUROSCI.0804-12.2012 PMC4235252

[ref72] Schairer KS, Ellison JC, Fitzpatrick D, Keefe DH. 2007. Wideband ipsilateral measurements of middle-ear muscle reflex thresholds in children and adults. J Acoust Soc Am. 121:3607–3616. 17552712 10.1121/1.2722213 PMC2041858

[ref73] Schofield BR, Beebe NL. 2019. Descending auditory pathways and plasticity. In: Kandler K, editor. The Oxford handbook of the auditory brainstem. New York, USA: Oxford University Press, pp. 610–638.

[ref74] Schroeder CE, Lakatos P. 2009. Low-frequency neuronal oscillations as instruments of sensory selection. Trends Neurosci. 32:9–18. 19012975 10.1016/j.tins.2008.09.012 PMC2990947

[ref75] Schubotz RI . 2007. Prediction of external events with our motor system: towards a new framework. Trends Cogn Sci. 11:211–218. 17383218 10.1016/j.tics.2007.02.006

[ref76] Singer Y, Teramoto Y, Willmore BD, Schnupp JW, King AJ, Harper NS. 2018. Sensory cortex is optimized for prediction of future input. elife. 7:e31557. 29911971 10.7554/eLife.31557 PMC6108826

[ref77] Smith DW, Aouad RK, Keil A. 2012. Cognitive task demands modulate the sensitivity of the human cochlea. Front Psychol. 3:30. 22347870 10.3389/fpsyg.2012.00030 PMC3277933

[ref78] Suga N, Ma X. 2003. Multiparametric corticofugal modulation and plasticity in the auditory system. Nat Rev Neurosci. 4:783–794. 14523378 10.1038/nrn1222

[ref79] Terreros G, Delano PH. 2015. Corticofugal modulation of peripheral auditory responses. Front Syst Neurosci. 9:134. 26483647 10.3389/fnsys.2015.00134 PMC4588004

[ref80] Todorovic A, van Ede F, Maris E, de Lange FP. 2011. Prior expectation mediates neural adaptation to repeated sounds in the auditory cortex: an MEG study. Neuroscience. 31:9118–9123. 21697363 10.1523/JNEUROSCI.1425-11.2011 PMC6623501

[ref81] Vallesi A, McIntosh AR, Shallice T, Stuss DT. 2009. When time shapes behavior: fMRI evidence of brain correlates of temporal monitoring. J Cogn Neurosci. 21:1116–1126. 18752413 10.1162/jocn.2009.21098

[ref82] Winer JA . 2005. Decoding the auditory corticofugal systems. Hear Res. 207:1–9. 16091301 10.1016/j.heares.2005.06.007

[ref83] Winer JA, Lee CC. 2007. The distributed auditory cortex. Hear Res. 229:3–13. 17329049 10.1016/j.heares.2007.01.017 PMC2637155

[ref84] Winkler I, Denham SL, Nelken I. 2009. Modeling the auditory scene: predictive regularity representations and perceptual objects. Trends Cogn Sci. 13:532–540. 19828357 10.1016/j.tics.2009.09.003

[ref85] Wittekindt A, Kaiser J, Abel C. 2014. Attentional modulation of the inner ear: a combined otoacoustic rmission and EEG study. J Neurosci. 34:9995–10002. 25057201 10.1523/JNEUROSCI.4861-13.2014 PMC6608308

[ref86] Wolpert S, Heyd A, Wagner W. 2014. Assessment of the noise-protective action of the olivocochlear efferents in humans. Audiol and Neurotol. 19:31–40. 10.1159/000354913 24281009

[ref87] Xiao Z, Suga N. 2002. Modulation of cochlear hair cells by the auditory cortex in the mustached bat. Nat Neurosci. 5:57–63. 11753417 10.1038/nn786

